# The pheromone-induced nuclear accumulation of the Fus3 MAPK in yeast depends on its phosphorylation state and on Dig1 and Dig2

**DOI:** 10.1186/1471-2121-8-44

**Published:** 2007-10-26

**Authors:** Ernest Blackwell, Hye-Jin N Kim, David E Stone

**Affiliations:** 1 Laboratory for Molecular Biology, University of Illinois at Chicago, Chicago, Illinois 60607, USA

## Abstract

**Background:**

Like mammalian MAP kinases, the mating-specific Fus3 MAPK of yeast accumulates in the nuclei of stimulated cells. Because Fus3 does not appear to be subjected to active nucleo-cytoplasmic transport, it is not clear how its activation by mating pheromone effects the observed change in its localization. One possibility is that the activation of Fus3 changes its affinity for nuclear and cytoplasmic tethers.

**Results:**

Dig1, Dig2, and Ste12 are nuclear proteins that interact with Fus3. We found that the pheromone-induced nuclear accumulation of a Fus3-GFP reporter is reduced in cells lacking Dig1 or Dig2, whereas Fus3^T180AY182A^-GFP localization was unaffected by the absence of these proteins. This suggests that Dig1 and Dig2 contribute to the retention of phosphorylated Fus3 in the nucleus. Moreover, overexpression of Ste12 caused the hyper-accumulation of Fus3-GFP (but not Fus3^T180AY182A^-GFP) in the nuclei of pheromone-treated cells, suggesting that Ste12 also plays a role in the nuclear retention of phosphorylated Fus3, either by directly interacting with it or by transcribing genes whose protein products are Fus3 tethers. We have previously reported that overexpression of the Msg5 phosphatase inhibits the nuclear localization of Fus3. Here we show that this effect depends on the phosphatase activity of Msg5, and provide evidence that both nuclear and cytoplasmic Msg5 can affect the localization of Fus3.

**Conclusion:**

Our data are consistent with a model in which the pheromone-induced phosphorylation of Fus3 increases its affinity for nuclear tethers, which contributes to its nuclear accumulation and is antagonized by Msg5.

## Background

Mitogen activated protein kinases (MAPKs) respond to a wide array of external signals, and are presumed to be present in all eukaryotes. Cellular proliferation, differentiation, and stress responses are all regulated by MAPKs [1-4]. The well characterized MAPKs function as part of a signaling module in which a MAPK kinase kinase (MEKK) phosphorylates and thereby activates a MAPK kinase (MEK), which then phosphorylates and activates the MAPK. Upon activation, MAPKs phosphorylate targets in the nucleus, in the cytoplasm, and at the cell cortex [5,6].

Because MAPKs affect targets throughout the cell, their localization is a critical aspect of signal regulation [7-9]. In fact, mislocalization of mammalian ERK MAPKs has been associated with altered signaling [10-13]. Moreover, the duration of ERK nuclear localization has been reported to be very important in determining the outcome of ERK activation: proliferation vs. differentiation [14-17]. Immunofluorescence and GFP-tagging experiments have demonstrated that pathway stimulation causes some MAPKs to translocate from the cytoplasm to the nucleus [18-20]. Although nuclear accumulation of MAPKs has been studied in model systems, the mechanisms that regulate this phenomenon are not fully understood.

*A priori*, the nuclear localization of MAPKs can be regulated in two ways: 1) regulated import or export across the nuclear membrane; 2) release from cytoplasmic tethers (anchoring proteins) and/or sequestration by nuclear tethers. Thus far, there are four examples of MAPKs that are subjected to active transport. In mammals, Erk2 interacts directly with the nuclear pore complex and undergoes Ran-dependent nuclear import [21-23], whereas Erk3 and Erk5 are actively exported from the nucleus by the CRM1 exportin [24,25]. In budding yeast, the Hog1 MAPK undergoes both Ran- and Nmd5-dependent nuclear import, as well as Crm1-dependent nuclear export [26]. A number of reports also suggest that tethers influence the localization of mammalian MAPKs. MEK is thought to be a cytoplasmic tether for ERK1/2, for example [27]. When ERK is activated, it releases from MEK, allowing ERK to localize to the nucleus. PEA-15, hSef, the MKP-3 dual-specific phosphatase, and the tyrosine phosphatases HePTP and PTP-SL have also been reported to be cytoplasmic tethers for ERKs, and β-arrestin is thought to anchor ERK to endosomal vesicles [28-34]. As yet, DUSP5 is the only protein reported to tether ERKs in the nucleus [35]. In the budding yeast *S.cerevisiae*, the Ptp2 phosphatase and the Msn2, Msn4, and Hot1 transcription factors act as nuclear tethers for the Hog1 MAPK, whereas the Ptp3 phosphatase and the Pbs2 MEK tether Hog1 in the cytoplasm [36-38]. Also in budding yeast, Spa2 retains the Mpk1 MAPK at sites of polarized growth [39]. Finally, the Atf1 transcription factor of *S.pombe *has been shown to regulate the nuclear localization of the Spc1/Sty1 MAPK [40].

To better understand the mechanisms controlling MAPK localization, we are using the mating reaction of *S.cerevisiae *as a model signaling system. When haploid yeast cells of opposite mating type are mixed, they undergo a complex mating reaction leading to the formation of zygotes. Each mating type constitutively secretes a peptide mating pheromone that triggers cells of the opposite type to arrest in the G1 phase of the cell cycle, form mating projections (shmoos), and induce mating-specific genes in preparation for cell and nuclear fusion. The signal to mate is transmitted across the plasma membrane by a seven transmembrane domain receptor and its associated heterotrimeric G protein. Upon release from Gα (Gpa1), the Gβγ dimer binds to the Ste5 scaffolding protein and stimulates a Pak kinase. These proteins, in turn, are required for activation of the MAP kinase cascade. The MAP kinase module consists of Ste11 (the MEKK), Ste7 (the MEK), and Fus3 (the MAPK). Upon activation by Ste7 on the Ste5 scaffold, the Fus3 MAPK accumulates at its sites of action [41-43]. In the nucleus, Fus3 phosphorylates the Ste12 mating-specific transcription factor [44,45], its two negative regulators, Dig1 and Dig2 [44,46], and a regulator of cell division, Far1 [44,45,47]. At the cortex, Fus3 is thought to phosphorylate Bni1 [48] and Gβ [49].

In proliferating cells, Fus3 is found in both the cytoplasm and the nucleus, with a slightly greater concentration of Fus3 in the nucleus [50,51]. Upon pheromone stimulation, the relative proportion of Fus3 in the nucleus increases. To quantify this pheromone-induced nuclear accumulation of Fus3, we developed an assay that measures the relative amount of Fus3 in each compartment [52]. Digital images of cells expressing a Fus3-GFP reporter were used to determine the ratio of nuclear to cytoplasmic fluorescence (RNCF). Using this assay, we confirmed that pheromone induces a measurable accumulation of Fus3 in the nucleus, and found that this increase in nuclear Fus3 correlates with the responsiveness of cells to pheromone. Cells that are resistant to pheromone-induced cell cycle arrest have lower RNCF values; cells that are hypersensitive to pheromone arrest have higher RNCF values. Moreover, the relative amount of Fus3 in the nucleus decreases as cells adapt to pheromone stimulation and re-enter the mitotic cycle. These results demonstrate that, although the changes in Fus3 localization during the induction and downregulation of the pheromone response are small (RNCF values typically range from 1.6 to 2.0), they are of great consequence to the cell. It is therefore of interest to determine what regulates the partitioning of Fus3 between the cytoplasm and the nucleus.

Using fluorescence recovery after photobleaching (FRAP) and fluorescence loss in photobleaching (FLIP) analyses, van Drogen and co-workers found no evidence that the rate of Fus3 transport either into or out of the nucleus is regulated by pheromone [51]. Rather, they concluded that Fus3 rapidly shuttles between the nucleus and cytoplasm by passive diffusion. If the rate of Fus3 transport across the nuclear membrane is not regulated by pheromone, then what causes the accumulation of Fus3 in the nuclei of pheromone-treated cells? One possibility is that the activation of Fus3 alters its affinity for cytoplasmic and nuclear tethering proteins. Consistent with this, Fus3 dissociates from the Ste5 scaffolding protein in the cytoplasm when it is phosphorylated by Ste7 [53]. Combining this mechanism with an increased tendency to bind tethers in the nucleus could account for the observed signal-induced change in Fus3 localization.

Here we report that three proteins known to interact with Fus3 in the nucleus (Dig1, Dig2, and Ste12) contribute to its pheromone-induced redistribution. Our data suggest that Dig1 and Dig2 are nuclear tethers for Fus3, and that Ste12 influences Fus3 localization either by directly interacting with it, or by transcribing genes whose protein products are Fus3 tethers. We also provide evidence that the phosphorylation state of Fus3 is a key determinant of its localization, and that the Msg5 phosphatase regulates Fus3 localization by dephosphorylating it in both the cytoplasm and in the nucleus. This work supports the idea that the activation of MAPKs changes their affinity for anchoring proteins in the cytoplasm and the nucleus, thereby effecting their signal-induced redistribution.

## Results

### Dig1 and Dig2 are required for the normal accumulation of Fus3 in the nuclei of pheromone-treated cells

**Figure 1:**
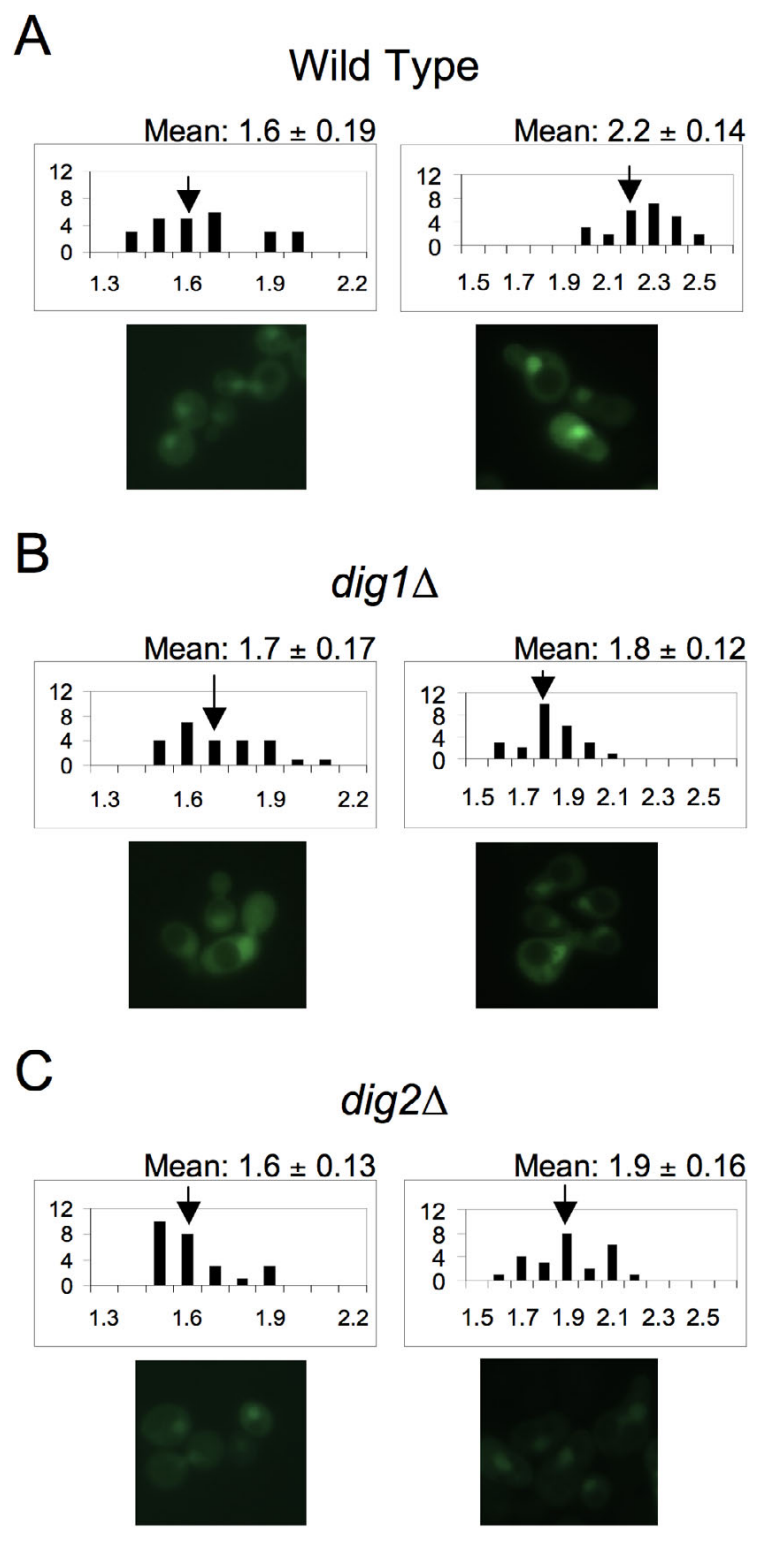
**Dig1 and Dig2 are required for the pheromone-induced nuclear accumulation of Fus3**. Strains of the indicated genotype were transformed with Fus3-GFP and grown to mid-log phase. The cultures were then split and grown with or without the addition of 12 nM pheromone. Images were acquired from the treated and untreated cells three hours later. The RNCF values were determined as described in the Materials and Methods, and their distributions represented in histograms. The number of cells (*y *axis) is plotted as a function of the RNCF values (*x* axis). Mean RNCF values are indicated ± s.d. In each panel, the untreated and treated cultures are represented by the left and right histograms, respectively. Arrows indicate the mean RNCF value for each culture. Representative micrographs from which the quantitative data were derived are shown beneath each histogram. (A)Wild type cells; (B) *dig1*Δ cells; (C)*dig2*Δ cells.

Dig1 and Dig2 are nuclear proteins that are known to interact with both the inactive and active forms of Fus3, with results from pull-down, two-hybrid, and peptide array binding experiments suggesting that these interactions are direct [46,54]. They negatively regulate the pheromone pathway by binding non-overlapping regions of Ste12 [55,56], a transcription factor essential for the expression of mating-specific genes [57-59]. To determine whether either Dig1 or Dig2 affects the nuclear localization of Fus3, we measured the cytoplasmic and nuclear distribution of Fus3-GFP in wild-type, *dig1*Δ, and *dig2*Δ cells. The distribution of the reporter was not significantly different in the three strains during vegetative growth (1.7 ± 0.17 vs. 1.6 ± 0.19 for *dig1*Δ vs. wild type, ρ = 0.33; 1.6 ± 0.13 vs. 1.6 ± 0.19 for *dig2*Δ vs. wild type, ρ = 0.40; compare the histograms in Fig. 1). In response to pheromone treatment, however, the wild-type cells exhibited a significantly higher concentration of Fus3 in the nucleus as compared to the mutant cells (1.8 ± 0.12 vs. 2.2 ± 0.14 for *dig1*Δ vs. wild type, ρ < 0.0001; 1.9 ± 0.16 vs. 2.2 ± 0.14 for *dig2*Δ vs. wild type, ρ < 0.0001; compare the histograms in Fig. 1). These data suggest that Dig1 and Dig2 contribute to the nuclear accumulation of Fus3 during the mating response, but either do not affect the localization of Fus3 in vegetative cells, or are functionally redundant in this regard.

Because the effect of *dig1*Δ and *dig2*Δ on Fus3-GFP localization is only detectable after pheromone treatment, and because pheromone treatment induces the phosphorylation/activation of Fus3, we wondered whether Dig1 and Dig2 specifically affect the localization of the phosphorylated form of Fus3. In stimulated cells, the pheromone-responsive MEK, Ste7, phosphorylates Fus3 on two residues, T180 and Y182 [60]. To determine the effect of Fus3 phosphorylation on its putative nuclear tethering by Dig1 and Dig2, we used a mutant form of the Fus3 reporter that cannot be phosphorylated by Ste7, Fus3^T180AY182A^-GFP [52]. The mutant reporter localized normally in vegetative wild type cells, but was defective in pheromone-induced nuclear accumulation (2.0 ± 0.14 vs. 2.2 ± 0.14 for Fus3^T180AY182A^-GFP vs. Fus3-GFP, ρ < 0.0001). Significantly, *dig1*Δ and *dig2*Δ had no measurable effect on the localization of the Fus3^T180AY182A^-GFP reporter (Table 1 - see additional file 1 for RNCF calculations). The insensitivity of Fus3^T180AY182A^-GFP localization to deletion of *DIG1 *and *DIG2 *suggests that Dig1 and Dig2 primarily tether the phosphorylated form of Fus3.

**Table 1:** RNCF and ρ values

	-	+ ^*a*^	ρ value ^*b*^	ρ value ^*c*^
control (Fus3-GFP)	1.6 ± 0.19	2.2 ± 0.14	n/a	n/a
*Dig1*Δ Fus3-GFP	1.7 ± 0.17	1.8 ± 0.12	0.3323	<0.0001
*Dig2*Δ Fus3-GFP	1.6 ± 0.13	1.9 ± 0.16	0.4017	<0.0001

				

control (Fus3^T180AY182A^-GFP)	1.6 ± 0.18	2.0 ± 0.14	n/a	n/a
*dig1*Δ Fus3^T180AY182A^-GFP	1.5 ± 0.16	2.0 ± 0.22	0.2722	0.1771
*dig2*Δ Fus3^T180AY182A^-GFP	1.5 ± 0.15	1.9 ± 0.16	0.0744	0.3527

				

control (empty vector)	1.6 ± 0.19	2.2 ± 0.14	n/a	n/a
Msg5 overexpression	1.4 ± 0.13	1.4 ± 0.20	0.0001	<0.0001
Msg5^M45A C319A ^overexpression	1.4 ± 0.12	1.7 ± 0.14	<0.0001	<0.0001

				

control (NLS-Msg5^M45A^/NES-	1.7 ± 0.17	1.9 ± 0.20	n/a	n/a
Msg5^M45A^)				
NLS-Msg5^M45A C319A^/NES-Msg5^M45A^	1.7 ± 0.18	2.1 ± 0.23	0.8380	0.0003
NLS-Msg5^M45A^/NES-Msg5^M45A C319A^	1.6 ± 0.18	2.1 ± 0.20	0.0490	0.0003

^*a*^RNCF ± s.d. 3 h after pheromone treatment; ^*b*^ρ value when compared to RNCF of untreated control; ^*c*^ρ value when compared to RNCF of pheromone-treated control. *n *= 25 for all experiments. See additional file 1 for histograms used to calculate the RNCF values for Table 1.

### Ste12 overexpression confers hyper-accumulation of Fus3 in the nuclei of pheromone-treated cells

**Figure 2:**
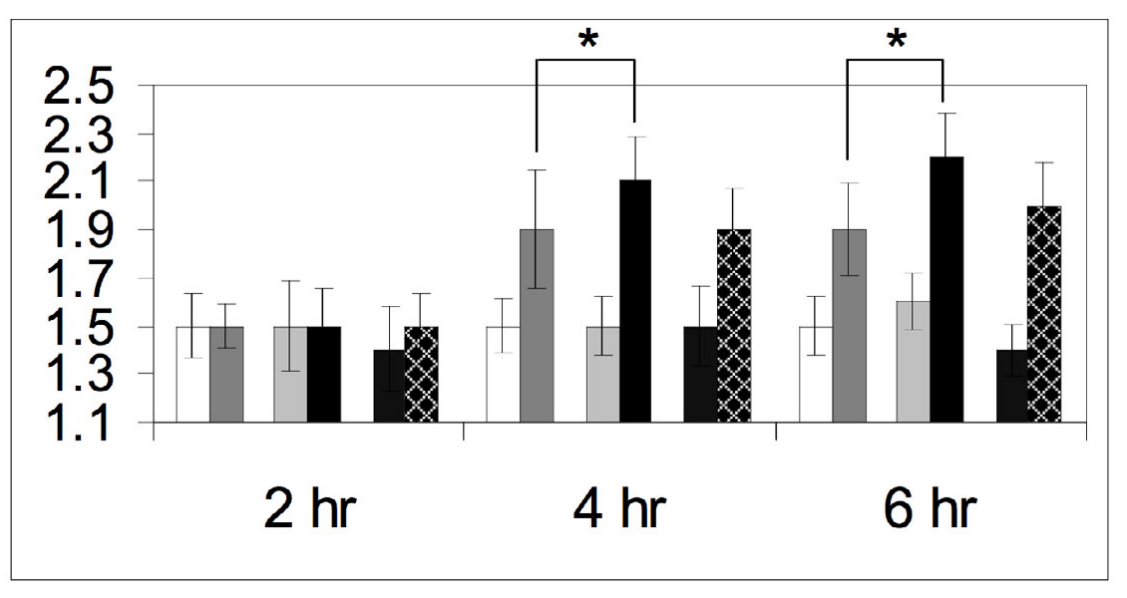
**Ste12 overexpression causes hyper-accumulation of Fus3 in the nuclei of pheromone-treated cells**. Wild type cells were transformed with either the Fus3-GFP reporter or the Fus3^T180AY182A^-GFP reporter, and either the *GAL1*-STE12 plasmid or an empty vector. Strains were grown to mid-log phase in sucrose medium and galactose was added to a concentration of 2% two hours before pheromone treatment (*GAL1 *promoter on). The cultures were then split and grown with or without the addition of 12 nM pheromone. Images were acquired from the untreated and treated cultures in 2 hour intervals. The mean RNCF ± s.d. values were determined as described in the Materials and Methods, and are represented in histograms as follows: wild type cells expressing Fus3-GFP–untreated (white bars) and treated (dark gray bars); wild type cells expressing Fus3-GFP and overexpressing Ste12–untreated (light gray bars) and treated (black bars); wild type cells expressing Fus3^T180AY182A^-GFP and overexpressing Ste12–untreated (stippled bars) and treated (hatched bars). Asterisks indicate statistically significant differences between strains with ρ = 0.003. See additional file 2 for images representative of each time point sampled for Figure 2.

The Ste12 transcription factor is required for the induction of many mating-specific genes [57-59]. It localizes to the nucleus [61] and, based on two-hybrid and biochemical data, is known to directly interact with Fus3 [45,62]. Therefore, like Dig1 and Dig2, Ste12 might be a nuclear tether for Fus3. To examine this possibility, we asked whether overexpression of Ste12 affects the nuclear accumulation of Fus3. We did not test the effect of *ste12*Δ on Fus3 localization because Ste12 is essential for normal activation of the pheromone pathway. A plasmid containing *STE12 *under the control of a galactose-inducible promoter was transformed into wild type cells carrying the Fus3-GFP reporter. Galactose-induced overexpression of Ste12 did not affect Fus3-GFP localization in vegetative cells (Fig. 2). However, when Ste12 overexpression was induced concomitant with the addition of pheromone, the pheromone-stimulated RNCF was significantly greater than in the control cells (2.1 ± 0.19 vs. 1.9 ± 0.25 for Ste12 overexpression vs. wild type, ρ = 0.0025; see the 4 hour time point in Fig. 2). Thus, excess Ste12 causes hyper-accumulation of Fus3-GFP in the nuclei of pheromone-treated but not untreated cells. To determine whether this effect depends on the phosphorylation of Fus3, we repeated the experiment in cells expressing the Fus3^T180AY182A^-GFP reporter. As shown in Figure 2, overexpression of Ste12 had no effect on Fus3^T180AY182A^-GFP localization (1.9 ± 0.17 vs. 1.9 ± 0.25 for Ste12 overexpression vs. wild type, ρ = 0.74; see the 4 hour time point in Fig. 2), consistent with the idea that Ste12 augments the nuclear tethering of activated Fus3 (see Additional file 2 for further details).

### Msg5 affects the nuclear accumulation of Fus3 by dephosphorylating it in both the cytoplasm and the nucleus

**Figure 3:**
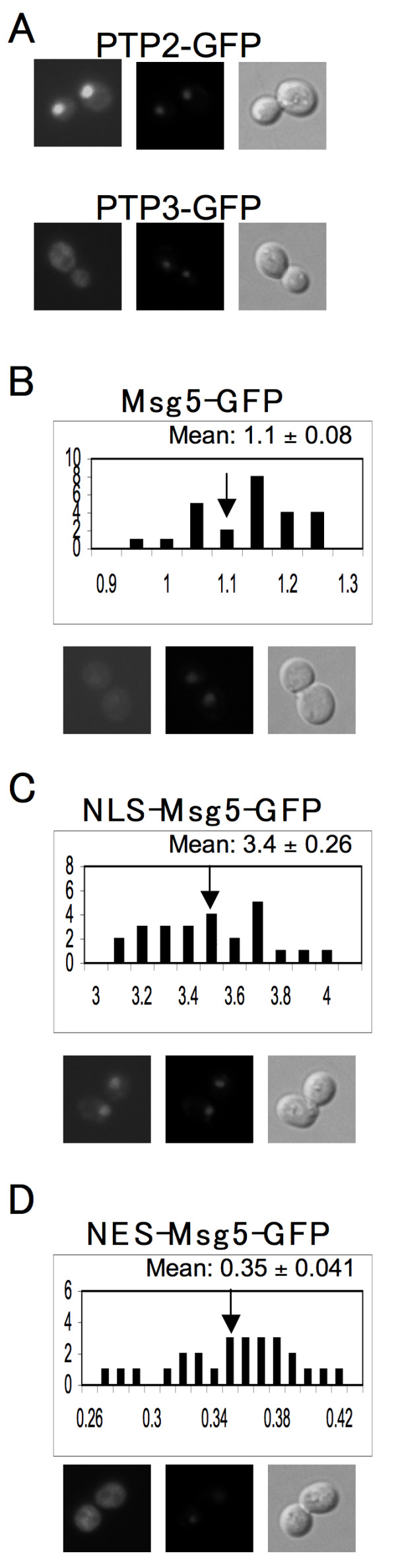
**Localization of the wild type, NLS-tagged, and NES-tagged forms of Msg5**. A set of 15Dau transformants carrying various centromeric reporter plasmids–PTP2-GFP, PTP3-GFP, MSG5-GFP, NLS-MSG5^M45A^-GFP, and NES-MSG5^M45A^-GFP–were grown to mid-log phase, stained with 20 μg/ml DAPI, and examined using fluorescent and differential interference contrast (DIC) microscopy. In each panel, representative images are displayed as follows: FITC (left), DAPI (middle), and DIC (right). RNCF values for Msg5-GFP, NLS-MSG5^M45A^-GFP, and NES-MSG5^M45A^-GFP were quantified in the same manner as for Fus3-GFP, and are represented as histograms. RNCF values are indicated as the mean ± s.d. (A)PTP2-GFP cells (top); PTP3-GFP cells(bottom); (B)MSG5-GFP cells; (C)NLS-MSG5^M45A^-GFP cells; (D)NES-MSG5^M45A^-GFP cells.

Msg5 is a pheromone-inducible, dual-specific phosphatase known to dephosphorylate and thereby inactivate Fus3 [63]. We have previously shown that overexpression of Msg5 lowers the amount of Fus3 found in the nucleus of vegetative cells [52]. In stimulated cells, excess Msg5 completely blocks the pheromone-induced nuclear accumulation of Fus3. Conversely, deletion of Msg5 confers hyper-accumulation of Fus3 in the nucleus [52]. Although these experiments strongly suggest that Msg5 affects the localization of Fus3, they do not tell us how. *A priori*, Msg5 could mediate its effect on Fus3 by tethering it in the cytoplasm and/or by dephosphorylating it on either side of the nuclear membrane. To investigate these possibilities, we first asked where Msg5 itself is located. An Msg5-GFP reporter was constructed and shown to be functional by its ability to compliment an *msg5*Δ allele in pheromone-response assays (data not shown). In vegetative cells, Msg5-GFP appeared to localize uniformly throughout the cytoplasm and nucleoplasm, and this pattern was unchanged after pheromone treatment (Fig. 3B). To determine whether the putative nuclear fluorescence was due to the presence of Msg5-GFP within the nucleus, and not simply around it, we compared cells expressing the Msg5 reporter to those expressing Ptp3-GFP. Ptp3 is a tyrosine phosphatase that inactivates Fus3 and its sister MAPK, Hog1 [64,65]. The Ptp3-GFP reporter has been shown to localize exclusively to the cytoplasm [38]. As shown in Fig. 3A, there is no detectable fluorescence in the nuclei of cells expressing Ptp3-GFP. This demonstrates that our imaging system can discern equal nuclear and cytoplasmic localization from purely cytoplasmic localization. Together, these results suggest that Msg5 is distributed evenly in both compartments, and that its localization does not change upon pheromone treatment.

Given the apparent distribution of Msg5 throughout the cytoplasm and the nucleoplasm, the effect of Msg5 on Fus3 nuclear localization can be explained in either of the two ways described above: Msg5 might tether Fus3 in the cytoplasm, just as Ptp3 is thought to tether Hog1 [38]. Alternatively, dephosphorylation of Fus3 by Msg5 might alter its affinity for anchoring proteins on either side of the nuclear membrane. Of course, these possibilities are not mutually exclusive.

To separate the potential abilities of Msg5 to regulate Fus3 by tethering and dephosphorylation, we created a phosphatase-dead form of the enzyme, Msg5^C319A^. The C319A substitution in Msg5 has been shown to specifically disrupt the catalytic activity of the phosphatase [63]. We then compared the localization of Fus3-GFP in wild-type cells and cells overexpressing either Msg5 or Msg5^C319A^. Excess Msg5^C319A ^lowered the basal RNCF as effectively as an excess of wild type Msg5 (Table 1). In contrast, Msg5^C319A ^was handicapped in its ability to regulate Fus3 localization in cells responding to pheromone: Although excess Msg5^C319A ^lowered the induced RNCF, the pheromone-stimulated nuclear accumulation of Fus3 was not completely blocked, as was the case in cells overexpressing wild-type Msg5 (Table 1). This suggests that the phosphatase activity of Msg5 is required for proper regulation of Fus3 localization during mating.

Does Msg5 inhibit the pheromone-induced change in RNCF by dephosphorylating Fus3 in the nucleus, the cytoplasm, or both? To answer this question, we fused either the nuclear localization signal (NLS) of the SV40 T antigen [66] or the nuclear export signal (NES) of HIV-1 [67] to the N-termini of *MSG5 *and *MSG5*^C319A^, preserving the native *MSG5 *promoter sequences. Because the *MSG5 *message has an alternate translational start site at codon 45 [68], we also engineered M45A substitutions in both constructs, thereby ensuring that all of the Msg5 protein encoded by the hybrid genes is N-terminally tagged. The resulting constructs are designated NLS-MSG5^M45A^, NES-MSG5^M45A^, NLS-MSG5^M45AC319A^, and NES-MSG5^M45A C319A^. All are contained on centromeric (i.e., low-copy) plasmids.

To determine the efficacy of the NLS and NES tags, we fused GFP to the C-termini of NLS-MSG5^M45A^, NES-MSG5^M45A^, NLS-MSG5^M45AC319A^, and NES-MSG5^M45A C319A^. Cells transformed with NLS-tagged Msg5 reporters exhibited dramatic nuclear concentration of the GFP signal, similar to that seen in cells expressing a GFP-tagged form of the nuclear phosphatase, Ptp2 (compare Figures 3A and 3C). RNCF measurements indicated that about 80% of the GFP-reporter signal was localized in the nuclei of cells expressing both NLS-Msg5^M45A^-GFP and NLS-MSG5^M45AC319A^-GFP. Cells transformed with NES-tagged Msg5 reporters exhibited dramatic cytoplasmic concentration of the GFP signal, similar to that seen in cells expressing a GFP-tagged form of the cytoplasmic phosphatase, Ptp3 (compare Figures 3A and 3D). RNCF measurements of the NES-tagged reporters indicated that approximately 70% of the reporter was localized to the cytoplasm.

By transforming an *msg5*Δ *Fus3-GFP *strain with various pairs of the NLS-tagged and NES-tagged Msg5^M45A ^and Msg5^M45AC319A ^centromeric plasmids, we sought to determine how Fus3 localization would be affected by loss of Msg5 phosphatase activity in one compartment or the other. Three strains were created: the NES-MSG5^M45A^/NLS-MSG5^M45A ^cells express enzymatically active Msg5 in both the cytoplasm and the nucleus; the NES-MSG5^M45AC319A^/NLS-MSG5^M45A ^cells express Msg5 in both compartments, but little or no phosphatase activity in the cytoplasm; and the NES-MSG5^M45A^/NLS-MSG5^M45AC319A ^cells express Msg5 in both compartments, with little or no phosphatase activity in the nucleus. Because all of the *MSG5 *alleles are expressed from the native *MSG5 *promoter, all are carried on low-copy plasmids from the YCplac series [69], and all were transformed into the same *msg5*Δ strain, the level and partitioning of Msg5 is very unlikely to vary significantly amongst the three strains in question. These three strains should differ only in where Msg5 is enzymatically active. As shown in Table 1, the basal RNCF values were not significantly different in the three strains, suggesting again that Msg5 phosphatase activity does not play a major role in regulating the localization of Fus3 in vegetative cells. In contrast, the pheromone-induced RNCF values were elevated in cells lacking full Msg5 phosphatase activity in either the cytoplasm or nucleus. This suggests that Msg5 negatively regulates the pheromone response by inactivating Fus3 both inside and outside of the nucleus.

**Figure 4:**
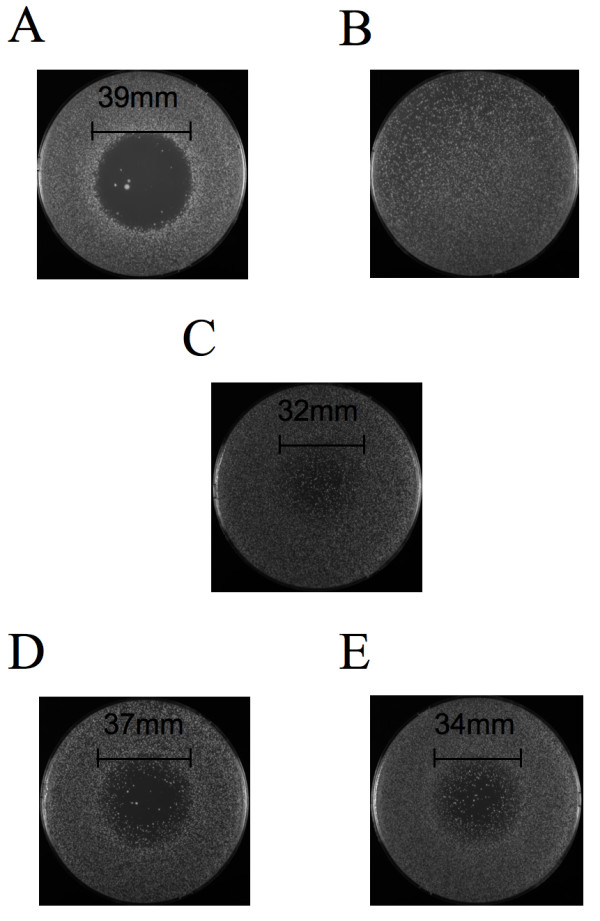
**Deficient Msg5 phosphatase activity in either the nucleus or cytoplasm confers increased sensitivity to pheromone**. Halo tests were performed as described in [83]. (A)Wildtype cells; (B) GAL-MSG5 cells; (C) NES-MSG5^M45A^/NLS-MSG5^M45A ^cells; (D)NES-MSG5^M45A^/NLS-MSG5^M45AC319A^; (E) NES-MSG5^M45AC319A^/NLS-MSG5^M45A ^cells.

We have previously established that the relative amount of Fus3-GFP in the nuclei of pheromone-treated cells correlates with sensitivity to pheromone-induced cell cycle arrest [52]. To determine whether the deficiency of Msg5 phosphatase activity and the consequent increase in the induced RNCF also corresponds to heightened sensitivity to pheromone, we performed halo tests. Compared to our wild type control strain, the three NES-MSG5/NLS-MSG5 strains all formed smaller halos that were partially filled in with pheromone resistant colonies (Fig. 4), a phenotype consistent with overexpression of Msg5 [63]. However, the three experimental strains were not equally responsive to pheromone in the halo tests. The cells deficient in cytoplasmic Msg5 activity (the NES-MSG5^M45AC319A^/NLS-MSG5^M45A ^cells) were more sensitive to pheromone-induced growth arrest than the cells with full Msg5 activity in both compartments (the NES-MSG5^M45A^/NLS-MSG5^M45A ^cells), and the cells deficient in nuclear Msg5 activity (the NES-MSG5^M45A^/NLS-MSG5^M45AC319A ^cells) were the most sensitive of all. This suggests that although Msg5 regulates pheromone-responsiveness in both the cytoplasm and the nucleus, the nuclear pool of Msg5 has a greater impact on the cell's sensitivity to pheromone-induced cell cycle arrest. These observations also strengthen the correlation between higher RNCF (more Fus3 in the nucleus) and greater cellular responsiveness to pheromone.

## Discussion

MAPK signaling modules are designed to relay signals originating at the plasma membrane to nuclear targets. This is accomplished by the phosphorylation and activation of the MAPK in the cytoplasm, followed by its accumulation in the nucleus. The signal-induced redistribution of the MAPK drives the nuclear responses, whereas these responses attenuate as the level of active MAPK in the nucleus declines. The kinetics with which MAPK nuclear activity rises and falls determines both the degree of response and, in some cases, the type of response [14-16]. For example, the decision of rat PC12 cells to proliferate or differentiate into neurites is thought to be controlled by how long Erk remains active in the nucleus after stimulation [70]. Clearly, the localization of MAPKs, like their enzymatic activity, must be tightly regulated.

Like Erk1/2, the mating-specific Fus3 MAPK of yeast regulates a developmental switch between proliferation and differentiation. Pheromone signaling increases the concentration of active Fus3 in the nucleus, which causes cell cycle arrest and induces the morphogenic changes necessary for efficient mating. The results reported by vanDrogen et al. [51] suggest that Fus3 diffuses freely into and out of the nucleus, at a rate unaffected by pheromone. If pheromone has no effect on the rate of Fus3 movement, then how does it increase the level of nuclear Fus3?

### Active Fus3 may be tethered by its nuclear substrates

One way to explain the pheromone-induced accumulation of nuclear Fus3 in the absence of regulated transport is differential tethering: Activation of Fus3 could cause it to dissociate from cytoplasmic tethers (e.g., Ste5) and bind to nuclear tethers. Although our experiments do not directly measure the level of active Fus3 in either compartment, our data are consistent with the idea that Fus3 is tethered by its nuclear substrates. Dig1 and Dig2 are substrates of Fus3, and each interacts directly with Fus3 in the nucleus. Deletion of either *DIG1 *or *DIG2 *conferred a defect in the accumulation of Fus3 in the nuclei of pheromone-treated cells but, even though Dig1 and Dig2 bind to the inactive form of Fus3 [46], had no significant effect on the nucleo-cytoplasmic partitioning of Fus3 in vegetative cells (Fig. 1). It is unlikely that the aberrant localization of Fus3 in pheromone-treated cells is due to a loss of Fus3 function, as *dig1*Δ and *dig2*Δ are reported to have no effect on Fus3 activity [46]. Therefore, the simplest explanation for our results is that Dig1 and Dig2 enhance retention of the phosphorylated/activated form of Fus3 in the nuclei of mating cells. Alternatively, deletion of *DIG1 *and *DIG2 *could affect the ratio of nuclear to cytoplasmic Fus3 indirectly by altering the expression of Ste12-regulated genes. Fus3, Msg5, and Dig2 are all known to be upregulated by Ste12 [71,72].

Another potential nuclear tether for Fus3 is the Ste12 transcription factor. Ste12 is a substrate of Fus3 and the two proteins directly interact in the nucleus. We found that overexpression of Ste12 significantly increased the nuclear accumulation of Fus3 but not of Fus3^T180AY182A^-GFP in pheromone-treated cells (Fig. 2), as would be expected if Ste12 specifically tethers the activated form of Fus3. It has been reported, however, that pheromone-treatment substantially diminishes the Fus3-Ste12 association, as assayed by co-IP [46]. Therefore, we favor the idea that Ste12 overexpression affects the nuclear accumulation of Fus3 indirectly, by hyper-inducing the expression of other nuclear tethers. Dig2 could be one such downstream tether, as its transcription is induced by pheromone [71]. Indeed, deletion of *DIG2 *lessened the effect of Ste12 overexpression on Fus3 localization, although the difference between the RNCF data sets was not quite statistically significant (data not shown). Unfortunately, we were not able to directly assess the effect of Ste12-induced transcription on Fus3 RNCF because the available transcription^- ^alleles of *STE12 *all contain large deletions, which could disrupt Ste12-Fus3 interaction. We conclude that Ste12 contributes to the retention of Fus3 in the nuclei of mating cells either by direct interaction with activated Fus3 or, more likely, by inducing the expression of other Fus3 nuclear anchoring proteins.

### The Msg5 phosphatase regulates Fus3 localization

The dual-specific Msg5 phosphatase inactivates Fus3 by removing the phosphates on residues T180 and Y182. In earlier work, we reported that overexpression of Msg5 had a dramatic effect on Fus3 localization: Excess Msg5 lowered the relative amount of nuclear Fus3 in vegetative cells and completely blocked its pheromone-induced nuclear accumulation [52]. Our discovery that Msg5 localizes homogenously to both the cytoplasm and the nucleoplasm (Fig. 3) is consistent with several means by which Msg5 might limit the pool of nuclear Fus3. Msg5 could sequester Fus3 in the cytoplasm, and/or it could dephosphorylate Fus3 in either or both compartments. To distinguish these possibilities, we examined the effects of altering Msg5 activity and localization on Fus3-GFP partitioning. We found that overexpression of Msg5^C319A^, an enzymatically dead form of the phosphatase, lowered the basal RNCF as effectively as the overexpressed wild type enzyme (Table 1). This indicates that Msg5 can sequester Fus3 in the cytoplasm. However, we do not believe that Msg5 tethering of Fus3 greatly influences Fus3 localization, as the Fus3:Msg5 protein ratio is about 16:1 in vegetative cells, and the pheromone-induction of *FUS3 *transcription is slightly greater than that of *MSG5 *[73].

A potentially more important finding is shown in Table 1. As compared to excess wild type Msg5, overexpressed Msg5^C319A ^was clearly defective in its ability to inhibit the nuclear accumulation of Fus3 in pheromone-treated cells. This implies that Msg5 phosphatase activity regulates the partitioning of Fus3 in mating cells. Given that Msg5 is overexpressed from the strong *GAL1 *promoter in these experiments, however, the extent to which dephosphorylation of Fus3 by Msg5 affects the localization of the kinase under physiological conditions was unclear. We were also interested to know whether Fus3 inactivation could be specifically attributed to the nuclear Msg5 phosphatase activity, as the cytoplasmic and nuclear pools of Msg5 could play distinct roles in Fus3 regulation. To address these questions, we constructed a set of three strains designed to express the same total amount of Msg5 in each compartment, but which are distinguished by whether they express Msg5 phosphatase activity in the cytoplasm, the nucleus, or both. Although Msg5 appears to be at least slightly overexpressed in these strains, as indicated by the halo tests (Fig. 4), the Msg5 levels are presumed to be more nearly normal than in the cells expressing *MSG5 *from the *GAL1 *promoter (compare the halo formed by the *GAL1-MSG5 *cells to the halos formed by the other strains). In essence, these strains allow us to ask what happens to the localization of Fus3 when Msg5 phosphatase activity is specifically turned off in either the nucleus or cytoplasm, while holding the total amount of Msg5 constant. Our data indicate that Fus3 hyper-localizes to the nuclei of pheromone-treated cells lacking Msg5 phosphatase activity in either compartment (Table 1). In contrast, loss of Msg5 phosphatase activity had no effect on the partitioning of Fus3 in vegetative cells (Table 1). These results strongly suggest that dephosphorylation/inactivation of Fus3 is an important means of regulating its localization in cells responding to pheromone, and that Msg5 performs this function in both the nucleus and cytoplasm.

Consistent with our previous results [52], the hyper-accumulation of Fus3 in the nuclei of Msg5 phosphatase-deficient cells correlated with increased sensitivity to pheromone-induced cell cycle arrest: The halos formed by cells lacking Msg5 phosphatase activity in the nucleus or cytoplasm were larger and less filled than those formed by the control cells (Fig. 4). Interestingly, loss of nuclear Msg5 phosphatase activity conferred a slightly more sensitive halo phenotype than loss of Msg5 phosphatase activity in the cytoplasm, suggesting that nuclear Msg5 has a greater impact on Fus3 regulation of the cell cycle than the cytoplasmic pool of the phosphatase.

A limitation of these Msg5-partitioning experiments is that we did not directly measure the nuclear and cytoplasmic levels of the various Msg5 forms. Although such measurements could strengthen our conclusions in principle, immunoblot-quantification of Msg5 after cell fractionation would not be sufficiently precise to be meaningfully correlated with the RNCF differentials that we observed. *A priori*, it is possible that the M45A or C319A substitutions in Msg5 could decrease its half-life. However, Flandez et al. have shown that myc-tagged Msg5^M45A ^is no less stable than the myc-tagged wild type protein [68], and the results of our Fus3-GFP localization assays argue strongly against the possibility that the C319A mutation destabilizes Msg5. As shown in Table 1, overexpression of Msg5^M45AC319A ^has the exact same impact on the basal RNCF as does the overexpression of Msg5, and expression of NES-Msg5^M45AC319A ^instead of NES-Msg5^M45A ^had no effect on the basal RNCFs. (If the C319A substitution conferred a significant decrease in the steady-state level of cytoplasmic Msg5, we would expect the basal RNCF values to increase, as is the case in *msg5*Δ cells [52]). Finally, even if we suppose that the steady-state level of NLS-Msg5^M45AC319A ^is lower than that of NLS-Msg5^M45A^, we would expect to find this difference in all the strains analyzed for Fig. 4 and the bottom section of Table 1. Instead of concluding that the Msg5 *phosphatase activity *in both the cytoplasm and nucleus inhibits pheromone-induced Fus3 nuclear localization, we would conclude simply that both cytoplasmic and nuclear Msg5 inhibits pheromone-induced Fus3 nuclear localization.

### Nucleo-cytoplasmic partitioning of Fus3–a switch and a timer

Considering our results with those of others, pheromone-induced nuclear accumulation can be explained quite simply as follows: Fus3 diffuses freely into and out of the nucleus at a rate that is unaffected by pheromone [51]. When the cell is exposed to pheromone, Fus3 is phosphorylated by Ste7, which causes it to shift to its active conformation. Activation of the kinase triggers its dissociation from Ste5 [53], while increasing its affinity for its nuclear targets. The interaction of Fus3 with its nuclear targets then retards its diffusion out of the nucleus. In short, Fus3 accumulates in the nucleus of pheromone-treated cells as the result of signal-induced changes in cytoplasmic and nuclear tethering. Fus3/Ste12-induced transcription of nuclear tethers (e.g., Dig2) would be expected to further increase the retention of Fus3 in the nucleus, thus creating a positive feedback loop. The fact that the absence of Dig1 or Dig2 did not affect the basal RNCF or completely inhibit the pheromone-induced increase in the RNCF suggests that there are other proteins involved in the nuclear tethering of Fus3.

Our results also suggest that Msg5 antagonizes the nuclear localization of Fus3. By removing the activating phosphates on Fus3, Msg5 decreases its affinity for its nuclear tethers. In addition to promoting adaptation to pheromone and return to the ground-state partitioning of Fus3 after cell fusion, this mechanism may play an important role in regulating the mating response. Looking at the system as a whole, the primary job of the MAPK is to report the status of the membrane-bound receptor to the nucleus. As long as Fus3 remains active, the cell is insensitive to changes in receptor signaling. Msg5 limits the duration of Fus3 activity, thereby ensuring that the kinase periodically re-associates with the Ste5 scaffold, where it receives updates about the upstream elements in the pathway. Thus, Msg5 is a timer. By continually reversing the Fus3 activation switch (phosphorylation), Msg5 sharpens the temporal connection between the stimulus and response.

If the nuclear accumulation of Fus3 is due to pheromone-induced tethering, as we suggest, then the flux of Fus3 from the nucleus to cytoplasm of cells responding to pheromone should decrease. Why was this change undetected when vanDrogen et al. tried to measure it? Most likely, the decrease in Fus3 "export" due to induced nuclear tethering is too small to be observed by FRAP analysis. The error in their measurement (~9%) is much greater than the rate change in Fus3 movement out of the nucleus that we would expect to see, assuming the nuclear accumulation of Fus3 is linear over time.

Another prediction of our model is that a mutant form of Fus3 that cannot be phosphorylated should be unable to accumulate in the nuclei of pheromone-treated cells. Indeed, the induced RNCF of the Fus3^T180AY182A^-GFP reporter is significantly lower than normal (Table 1). On the other hand, pheromone treatment of wild type cells expressing Fus3^T180AY182A^-GFP does induce some increase in RNCF. If the phosphorylation of Fus3 is essential to its nuclear tethering, then why does the Fus3^T180AY182A^-GFP reporter accumulate in the nuclei of pheromone-treated cells at all? One possibility is that Fus3^T180AY182A^-GFP dimerizes with wild type Fus3, and these heterodimers are retained in the nucleus by a subset of Fus3 targets. In support of this explanation, Fus3 interacts with itself in the two-hybrid assay [74], and the Erk MAPKs are known to dimerize in response to stimulus [75]. Although we have not tested Fus3 dimerization directly, one of our previously published observations is consistent with it: Pheromone induces the nuclear accumulation of the Fus3^T180AY182A^-GFP reporter in cells expressing either Fus3 or Kss1 (which also interacts with Fus3 in the two-hybrid assay), but not in cells lacking functional copies of both these MAPKs [52]. Alternatively, the phosphorylation of Fus3 may be necessary for its efficient tethering by some nuclear proteins (e.g., Dig1 and Dig2), but not others. In this view, the pheromone-induced nuclear accumulation of Fus3^T180AY182A^-GFP would require expression of functional Fus3 or Kss1 because tethering is augmented by the MAPK-dependent phosphorylation of some Fus3 anchoring proteins.

## Conclusion

Our findings suggest that phosphorylation of Fus3 augments its ability to interact with nuclear tethers, which explains the nuclear accumulation of Fus3 observed after pheromone pathway activation. Additionally, the Msg5 phosphatase counteracts the nuclear accumulation of Fus3 by dephosphorylating it in both the nucleus and the cytoplasm. The continual dephosphorylation of Fus3 throughout the cell may enhance the temporal sensitivity of the pheromone pathway, thereby promoting a rapid adjustment of signalling intensity in response to changes in the pheromone gradient.

## Methods

### Molecular and microbiological techniques

Recombinant DNA techniques were essentially as described by Sambrook et al. [76] and by Ausubel et al. [77].

Yeast transformations were carried out according to the method of Ito et al. [78]. Yeast growth media were prepared as described by Sherman et al. [79]. Amino acids were omitted as necessary to select for plasmids. For experiments requiring the induction of the *GAL1*-regulated genes, cells were either grown to mid-log phase in sucrose medium and galactose added to a final concentration of 2%, or the cells were cultured overnight in medium containing galactose. The yeast strains used in this study are listed in Table 2.

**Table 2:** *S. cerevisiae *strains used in this study

*Strain*	*Relevant genotype*	*Source*
15Dau	*MAT***a ***bar1*Δ *ade1 his2 leu2–3,112 trp1 ura3*Δ	[80]
IHY165	*MAT***a ***bar1*Δ *ade1 his2 leu2–3,112 trp1 ura3*Δ *fus3*Δ::*LEU2 kss1*Δ::*KanMx*	This study
TBY281	*MAT***a ***bar1*Δ *ade1 his2 leu2–3,112 trp1 ura3*Δ *fus3*Δ::*LEU2 dig1*Δ::*KanMx*	This study
TBY287	*MAT***a ***bar1*Δ *ade1 his2 leu2–3,112 trp1 ura3*Δ *fus3*Δ::*LEU2 dig2*Δ::*KanMx*	This study
HKY137	*MAT***a ***bar1*Δ *ade1 his2 leu2–3,112 trp1 ura3*Δ *msg5*Δ::*LEU2*	This study

### Plasmids and strains

All strains used in the present study were derived from strain 15Dau (*MAT***a ***bar1*Δ *ade1 his2 leu2–3,112 trp1 ura3*Δ), which is isogenic with strain BF264-15D [80]. Deletion mutations were created by transformation with disruption plasmids and PCR products containing the yeast markers *URA3 *and *LEU2 *and the bacterial markers KanMx and HISG (Table 3). KanMx deletion cassettes were also amplified from the yeast deletion library [81]. Disruptions were confirmed by genomic PCR analysis and by pheromone response assays.

**Table 3:** Plasmids used in this study

*Plasmid*	*Marker*	*Source*
pIH4.1	URA3 CEN FUS3-GFP	[52]
pIH4.1AA	URA3 CEN FUS3^T180AY182A^-GFP	[52]
YCplac22/FUS3-GFP (AOB117)	TRP1 CEN FUS3-GFP	[52]
YCplac22/FUS3^T180AY182A^-GFP (TBB182)	TRP1 CEN FUS3^T180AY182A^-GFP	[52]
pRS316CG/MSG5-GFP (HKB101)	URA3 CEN MSG5-GFP	[52]
YCplac111/GAL1-MSG5 (HKB100)	LEU2 CEN GAL1-MSG5	[52]
YCplac22/GAL1-MSG5 (AEB101)	TRP1 CEN GAL1-MSG5	[52]
YCplac111/GAL1-MSG5^C319A ^(TBB109)	LEU2 CEN GAL1-MSG5^C319A^	This study
YCplac22/GAL1-STE12 (TBB157)	TRP1 CEN GAL1-STE12	This study
YCplac22/NLS-MSG5^M45A ^(TBB172)	TRP1 CEN NLS-MSG5^M45A^	This study
YCplac22/NES-MSG5^M45A ^(TBB174)	TRP1 CEN NES-MSG5^M45A^	This study
YCplac33/NLS-MSG5^M45A ^(TBB181)	URA3 CEN NLS-MSG5^M45A^	This study
YCplac33/NES-MSG5^M45A ^(TBB180)	URA3 CEN NES-MSG5^M45A^	This study
YCplac22/NLS-MSG5^M45AC319A ^(TBB178)	TRP1 CEN NLS-MSG5^M45AC319A^	This study
YCplac22/NES-MSG5^M45AC319A ^(TBB176)	TRP1 CEN NES-MSG5^M45AC319A^	This study
pRS316CG/NLS-MSG5-GFP^M45A ^(TBB171)	URA3 CEN NLS-MSG5^M45A^-GFP	This study
pRS316CG/NES-MSG5-GFP^M45A ^(TBB170)	URA3 CEN NES-MSG5^M45A^-GFP	This study
YEplac181/PTP2-GFP	LEU2 2 μ PTP2-GFP	[84]
YEplac181/PTP3-GFP	LEU2 2 μ PTP3-GFP	[84]

The plasmids used in this study are listed in Table 3. The construction of those that were created for this investigation is described below. All mutations were introduced using the QuikChange site-directed mutagenesis kit (Stratagene, La Jolla, CA), and all mutagenized products were sequenced by either Macrogen (Seoul, Korea) or Functional Biosciences, Inc. (Madison, WI).

#### (i) GAL-MSG5^C319A ^(TBB109)

The phosphatase dead version of Msg5 was created using the *GAL1-MSG5 *plasmid HKB100 [52] as a template. The primers used to introduce the C319A mutation were 5'-CTCGTACACGCTCAGTGTGG AGTATCAAGA TCGG-3' and 5'-CCGATCTTGA TACTCCACAC TGAGCGTGTA CGAG-3'. The substitutions are underlined.

#### (ii) GAL1-STE12 (TBB157)

The 0.680 kb *Bam*HI-*Eco*RI fragment containing the divergent *GAL1*/*GAL10 *promoter was moved from YCpG2 [82] to the polylinker of YCplac22 [69] to create plasmid ZWB159. The *STE12 *coding region (-20 to 2200) was amplified from genomic DNA by using the following primers: 5'-TGACCAAGCTTGAATTGTCT TGTTCACCAA GG-3', which contains a *HindIII *site (underlined), and 5'-AAACTGCAGG CTCTAGTCTT GTATAAGATT G-3', which contains a *PstI *site (underlined). The *STE12 *PCR product was then digested with *HindIII *and *PstI*, and ligated into *HindIII*-*PstI*-cut ZWB159 to create the *GAL1*-*STE12 *transcriptional fusion vector.

#### (iii) NLS-MSG5^M45A ^(TBB172, TBB181) and NES-MSG5^M45A ^(TBB174, TBB180)

The *MSG5 *coding region and promoter (-381 to 1470) were amplified from genomic DNA using the following primers: 5'-CCAACTGCAG ATGGTCCATC CTGGTAAG-3', which contains a *PstI *site (underlined), and 5'-GCGCGGATCC TTAAGGAAGA AACATCATCT GTT-3', which contains a *BamHI *site (underlined). The *MSG5 *PCR product was then digested with *PstI *and *BamHI*, and ligated into *PstI-BamHI*-cut YCplac22 [69]. To introduce the M45A mutation, the resulting MSG5/YCplac22 plasmid was amplified using the following primers: 5'-GGCTTGGCGA TGAGAATTCC GCTAATGGAT GGAGTGCTGC-3' and 5'-GCAGCACTCC ATCCATTAGC GGAATTCTCA TCGCCAAGCC-3'. The mutations are underlined. The resulting MSG5^M45A ^plasmid was then used as a template in two mutagenic PCR reactions designed to insert either the NLS sequence [66] or the NES sequence [67] immediately after the *MSG5 *start codon. The NLS tag was added using the following primers: 5'-GGTAGCGATA AGTGCACATG CCGAAGAAGAAGCGCAAGGTGCAATTTCAC TCAGATAAGC AGC-3' and 5'-GCTGCTTATC TGAGTGAAAT TGCACCTTGCGCTTCTTCTTCGGCATGTGC ACTTATCGCT ACC-3'. The NLS region is underlined. The NES tag was added using the following primers: 5'-GGTAGCGATA AGTGCACATG CTCCAGCTCCCACCACTCGAGCGACTCACACTCCAATTTC ACTCAGATAA GCAGC-3' and 5'-GCTGCTTATC TGAGTGAAAT TGGAGTGTGAGTCGCTCGAGTGGTGGGAGCTGGAGCATGT GCACTTATCG CTACC-3'. The NES region is underlined. The resulting plasmids, YCplac22 NLS-MSG5^M45A ^and YCplac22 NES-MSG5^M45A ^(TBB172 and TBB174), were digested with *BamHI *and *PstI*. The NLS-MSG5^M45A ^and NES-MSG5^M45A ^fragments were then purified and ligated into *PstI-BamHI*-cut YCplac33 to generate the desired YCplac33 NLS-MSG5^M45A ^and YCplac33 NES-MSG5^M45A ^plasmids (TBB181 and TBB180).

#### (iv) NLS-MSG5^M45A C319A ^(TBB178) and NES-MSG5^M45A C319A ^(TBB176)

To generate the C319A mutation in *MSG5*, plasmids TBB172 and TBB174 were used as templates. The mutagenic primers were: 5'-CTCGTACACGCTCAGTGTGG AGTATCAAGA TCGG-3' and 5'-CCGATCTTGA TACTCCACAC TGAGCGTGTA CGAG-3'. The substitutions are underlined.

#### (v) NLS-MSG5^M45A^-GFP (TBB171) and NES-MSG5^M45A^-GFP (TBB170)

The MSG5-GFP plasmid, HKY 101 [52], was used as a template for two PCR reactions designed to insert either the NLS sequence [66] or the NES sequence [67] immediately after the start codon of *MSG5*. The NLS was added using the following primers: 5'-GGTAGCGATA AGTGCACATG CCGAAGAAGAAGCGCAAGGTGCAATTTCAC TCAGATAAGC AGC-3' and 5'-CCATCGCTAT TCACGTGTAC GGCTTCTTCTTCGCGTTCCACGTTAAAGTG AGTCTATTCG TCG-3'. The NLS region is underlined. The NES was added using the following primers: 5'-GGTAGCGATA AGTGCACATG CTCCAGCTCCCACCACTCGAGCGACTCACACTCCAATTTC ACTCAGATAA GCAGC-3' and 5'-CCATCGCTAT TCACGTGTAC GAGGTCGAGGGTGGTGAGCTCGCTGAGTGTGAGGTTAAAG TGAGTCTATT CGTCG-3'. The NES region is underlined. Following isolation of the NLS-MSG5-GFP and NES-MSG5-GFP constructs, a 985 bp *ClaI-NsiI *fragment of *MSG5 *(46–1031 bp), was replaced in each with the corresponding fragment from TBB172, which contains the M45A mutation. The resulting plasmids were designated NLS-MSG5^M45A^-GFP and NES-MSG5^M45A^-GFP.

#### (vi) YCplac22/Fus3^T180A Y182A^-GFP (TBB182)

The *EcoRI-SacI *fragment containing the Fus3^T180AY182A^-GFP translational fusion was moved from IH4.1AA [52] to YCplac22 [69].

### Pheromone response halo tests

Strains were tested for pheromone-induced growth inhibition in standard halo assays as previously described [83].

### Photomicrographs and quantification of Fus3-GFP localization

Fluorescent and differential interference contrast (DIC) images were acquired using a Zeiss Axioskop 2 microscope fitted with a Zeiss AxioCam digital camera, and processed using Zeiss AxioVision software. Localization of Fus3-GFP was performed as described in [52]. Briefly, the Fus3-GFP signal was quantified from digital images by using the histogram function of Adobe Photoshop 5.5. A circle of set size was used to sample the brightness of the nucleus and cytoplasm of cells from randomly chosen fields. The ratio of nuclear to cytoplasmic fluorescence was rounded to the nearest 0.1, and values corresponding to at least 25 cells for each strain and condition were plotted in histograms. All such experiments were performed at least three times and individual experiments illustrating the general trend seen throughout all experiments are presented. Student's t-test was used for statistical analysis.

## Authors' contributions

EB constructed strains and plasmids, performed the Fus3 localization assays and halo tests, and drafted the manuscript in collaboration with DS. HK constructed strains and plasmids and collected the data presented in Fig. 3. DS conceived of the study and participated in its design and coordination. All authors read and approved the final manuscript.

## Supplementary Material

Additional file 1 RNCF calculations for strains listed in Table 1. Description: Strains of the indicated genotype were transformed with Fus3-GFP (A and E-I) or Fus3^T180AY182A^-GFP (B-D) and grown to mid-log phase. The cultures were then split and grown with or without the addition of 12 nM pheromone. Images were acquired from the treated and untreated cells three hours later. The RNCF values were determined as described in the Materials and Methods, and their distributions represented in histograms. The number of cells (*y *axis) is plotted as a function of the RNCF values (*x *axis). Mean RNCF values are indicated ± s.d. In each panel, the untreated and treated cultures are represented by the left and right histograms, respectively. Arrows indicate the mean RNCF value for each culture. (A)Wild type cells with Fus3-GFP; (B) Wild type cells with Fus3^T180AY182A^-GFP; (C) *dig1*Δ cells; (D)*dig2*Δ cells; (E) Msg5 overexpression cells; (F) MSG5^M45AC319A ^overexpression cells; (G) NES-MSG5^M45A^/NLS-MSG5^M45A ^cells; (H) NES-MSG5^M45A^/NLS-MSG5^M45AC319A ^cells; (I) NES-MSG5^M45AC319A^/NLS-MSG5^M45A ^cells. Click here for file

Additional file 2 Ste12 overexpression causes hyper-accumulation of Fus3 in the nuclei of pheromone-treated cells. Wild type cells were transformed with either the Fus3-GFP reporter or the Fus3^T180AY182A^-GFP reporter, and either the *GAL1*-STE12 plasmid or an empty vector. Strains were grown to mid-log phase in sucrose medium and galactose was added to a concentration of 2% two hours before pheromone treatment (*GAL1 *promoter on). The cultures were then split and grown with or without the addition of 12 nM pheromone. Images were acquired from the untreated and treated cultures in 2 hour intervals. The 2 hr, 4 hr, and 6 hr time points indicate time elapsed after galactose induction. (A) untreated wild type cells expressing Fus3-GFP; (B) pheromone-treated wild type cells expressing Fus3-GFP; (C) untreated cells expressing Fus3-GFP and overexpressing Ste12; (D) pheromone-treated cells expressing Fus3-GFP and overexpressing Ste12; (E) untreated cells expressing Fus3^T180AY182A^-GFP and overexpressing Ste12; (F) pheromone-treated cells expressing Fus3^T180AY182A^-GFP and overexpressing Ste12. Click here for file
