# Supplementary figures and images for: The pheromone-induced nuclear accumulation of the Fus3 MAPK in yeast depends on its phosphorylation state and on Dig1 and Dig2
Source: BMC Cell Biol. 2007 Oct 26;8:44. doi: 10.1186/1471-2121-8-44 (PMC2219999; doi:10.1186/1471-2121-8-44)

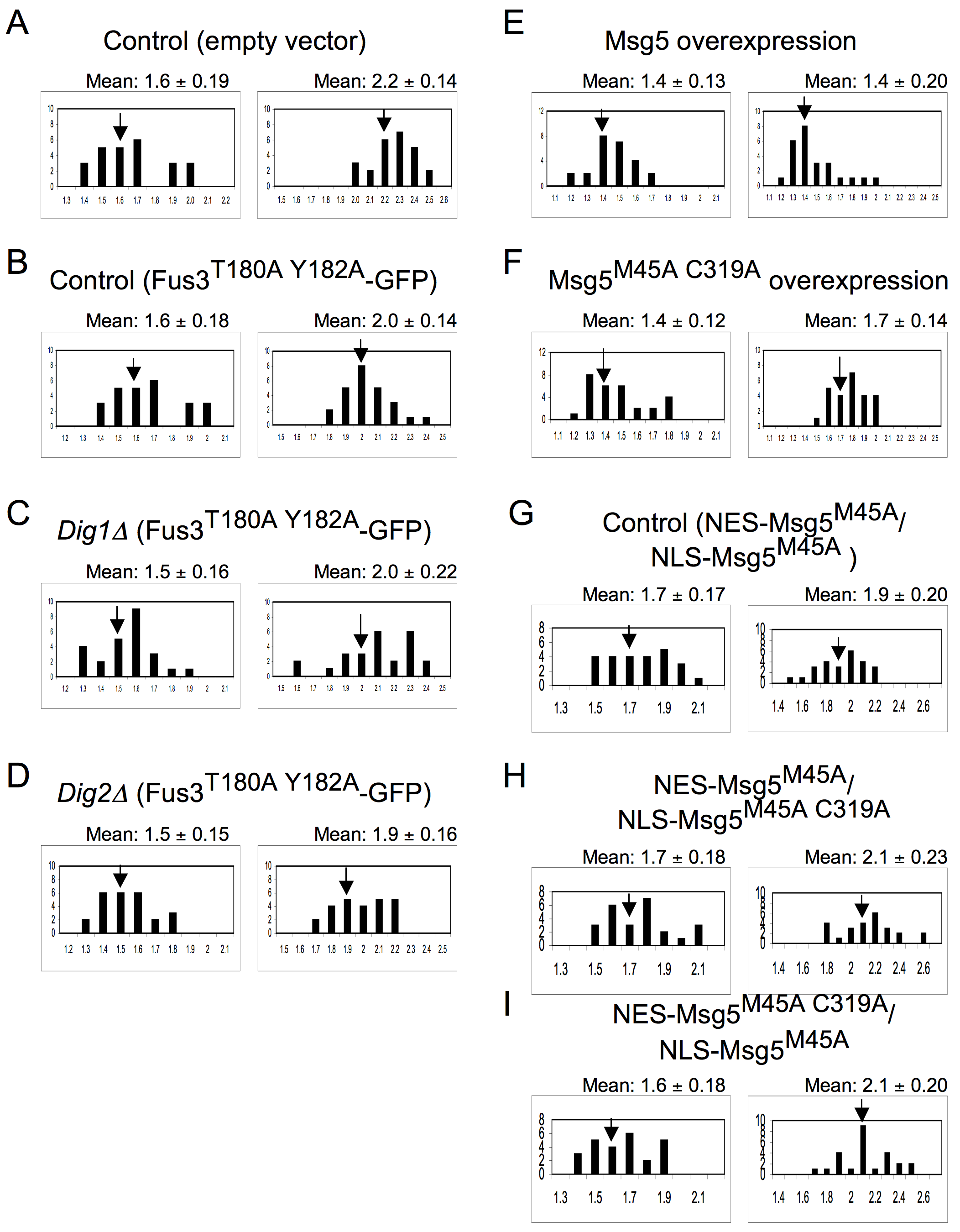

Supplement: Additional file 1 — RNCF calculations for strains listed in Table 1. Description: Strains of the indicated genotype were transformed with Fus3-GFP (A and E-I) or Fus3T180AY182A-GFP (B-D) and grown to mid-log phase. The cultures were then split and grown with or without the addition of 12 nM pheromone. Images were acquired from the treated and untreated cells three hours later. The RNCF values were determined as described in the Materials and Methods, and their distributions represented in histograms. The number of cells (y axis) is plotted as a function of the RNCF values (x axis). Mean RNCF values are indicated ± s.d. In each panel, the untreated and treated cultures are represented by the left and right histograms, respectively. Arrows indicate the mean RNCF value for each culture. (A)Wild type cells with Fus3-GFP; (B) Wild type cells with Fus3T180AY182A-GFP; (C) dig1Δ cells; (D)dig2Δ cells; (E) Msg5 overexpression cells; (F) MSG5M45AC319A overexpression cells; (G) NES-MSG5M45A/NLS-MSG5M45A cells; (H) NES-MSG5M45A/NLS-MSG5M45AC319A cells; (I) NES-MSG5M45AC319A/NLS-MSG5M45A cells. [file 1471-2121-8-44-S1.png]

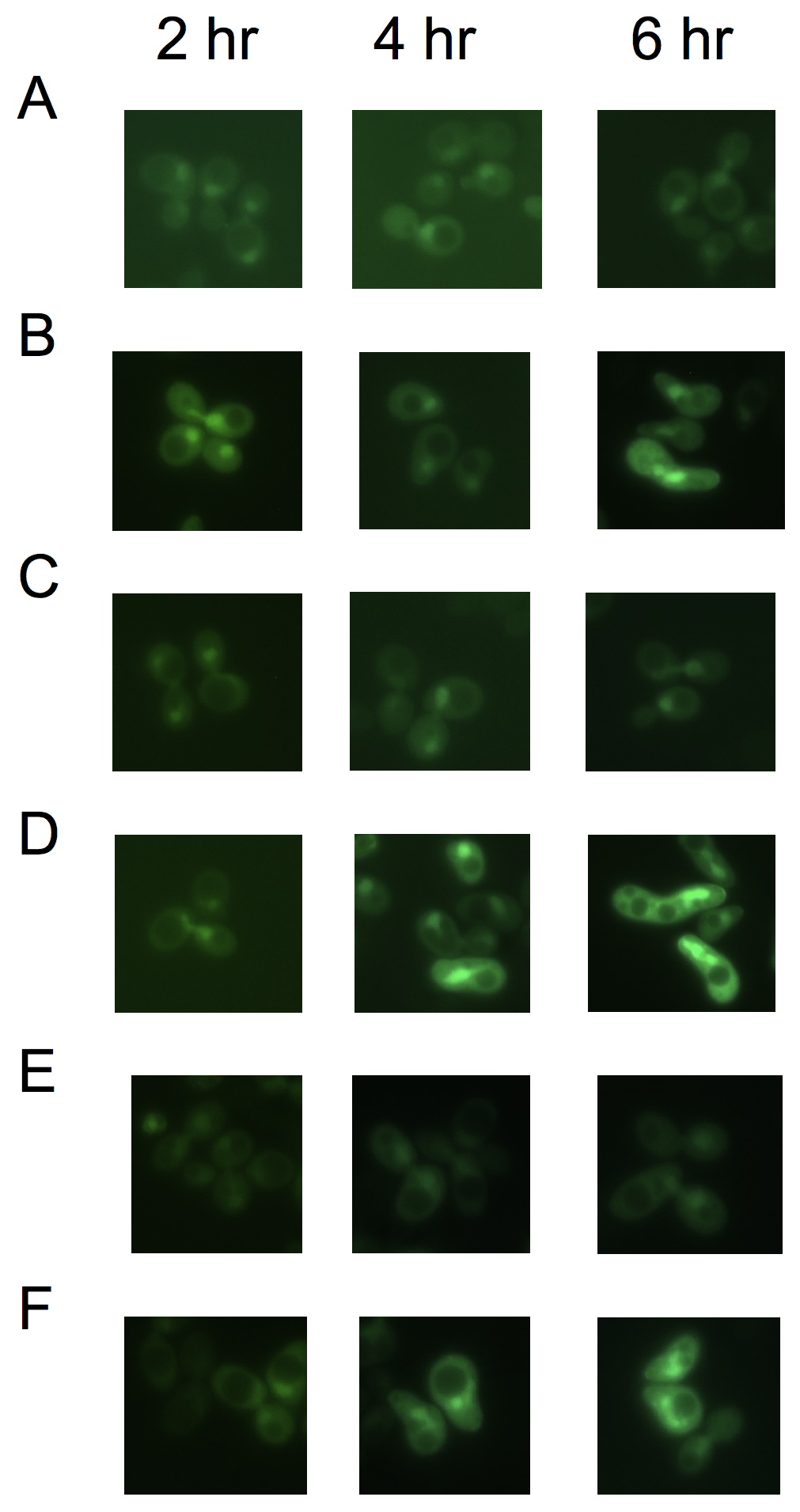

Supplement: Additional file 2 — Ste12 overexpression causes hyper-accumulation of Fus3 in the nuclei of pheromone-treated cells. Wild type cells were transformed with either the Fus3-GFP reporter or the Fus3T180AY182A-GFP reporter, and either the GAL1-STE12 plasmid or an empty vector. Strains were grown to mid-log phase in sucrose medium and galactose was added to a concentration of 2% two hours before pheromone treatment (GAL1 promoter on). The cultures were then split and grown with or without the addition of 12 nM pheromone. Images were acquired from the untreated and treated cultures in 2 hour intervals. The 2 hr, 4 hr, and 6 hr time points indicate time elapsed after galactose induction. (A) untreated wild type cells expressing Fus3-GFP; (B) pheromone-treated wild type cells expressing Fus3-GFP; (C) untreated cells expressing Fus3-GFP and overexpressing Ste12; (D) pheromone-treated cells expressing Fus3-GFP and overexpressing Ste12; (E) untreated cells expressing Fus3T180AY182A-GFP and overexpressing Ste12; (F) pheromone-treated cells expressing Fus3T180AY182A-GFP and overexpressing Ste12. [file 1471-2121-8-44-S2.png]
